# Recommended protocol for performing oral fundus fluorescein angiography (FFA) in children

**DOI:** 10.1038/s41433-020-01328-6

**Published:** 2020-12-15

**Authors:** Oliver R. Marmoy, Robert H. Henderson, Kuan Ooi

**Affiliations:** 1 Clinical and Academic Department of Ophthalmology, Great Ormond Street Hospital for Children, London, UK; 2 UCL-GOS Institute of Child health, London, UK; 3 Manchester Metropolitan University, Manchester, UK; 4 Pharmacy Department, Great Ormond Street Hospital for Children, London, UK

**Keywords:** Eye abnormalities, Blood flow

## To the Editor:

Fundus Fluorescein Angiography (FFA) is a specialist ophthalmic test to visualise the retinal vasculature. FFA is conventionally performed through intravenous injection of 10–20% Sodium Fluorescein dye, following which serial retinal imaging (~490 nm wavelength) is used to dynamically visualise retinal flow, circulation and vascular integrity. FFA is a clinically valuable test in many conditions, in particular those causing non-perfusion, leakage, oedema, or other vascular anomalies [1].

As FFA typically requires intravenous injection of Fluorescein, this procedure is less tolerated by children and may have to be performed under anaesthesia. Furthermore, use of needles in children may have adverse psychological effects, which is a major consideration for children with chronic conditions. Intravenous FFA holds a moderate risk of adverse effects, rarely serious, while oral FFA has shown no serious adverse effects in large cohorts [2]. However, the seminal studies describing this technique in children used low-doses and therefore image acquisition times range from 15–60 min [3, 4]. We describe a protocol for performing oral FFA in children, which our department has utilised with great success, where image acquisition times typically last <30 min.

**Fig. 1:**
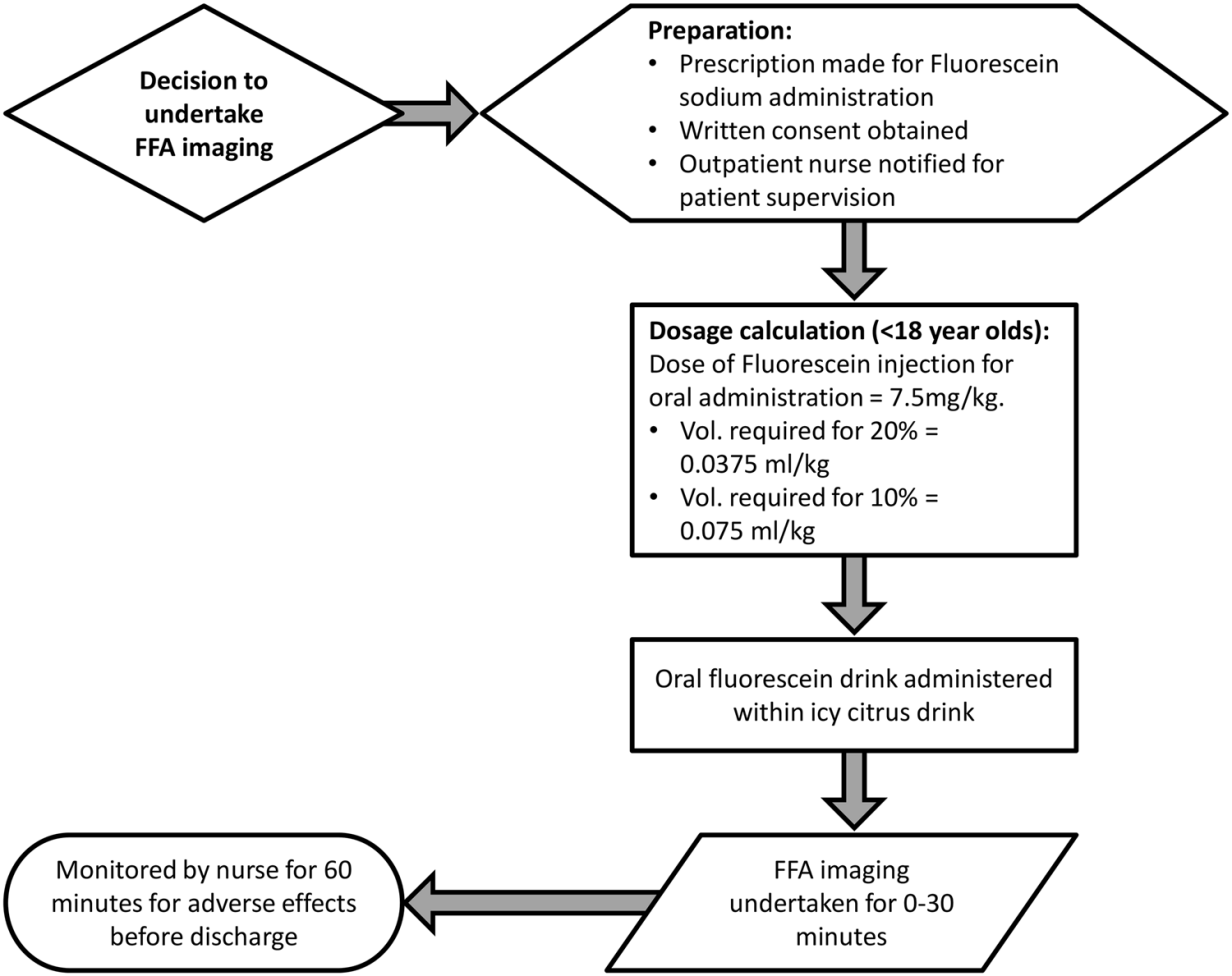
Protocol flow chart for performing oral FFA imaging in children. Once a decision is made to perform FFA imaging, the preparation phase begins for clinician prescription of Fluorescein Sodium, written consent/assent to be obtained from the patient and nursing staff to be notified to supervise the patient. The dosage calculation is made according to agreed locally agreed values for children. The child is then asked to consume the Fluorescein, which is mixed into an icy cold citrus drink such as orange or apple juice. The FFA imaging then begins, lasting a maximum of 30 min for most patients. Following the test, the patient must remain on site until an hour after the dose was administered to monitor for any adverse effects before leaving.

We outline our flow chart for the oral FFA technique in children in Fig. 1. Once a decision has been made to have an FFA examination, the patient is instructed on the day of the test to have a light breakfast without lunch before the study. Written consent or assent is obtained from the patient.

A 20% Fluorescein sodium solution dosage is calculated and the patient is provided a straw to consume the drink as fast as is comfortable for them, with special care taken to ensure the drink is not swirled within the mouth resulting in temporary tooth staining. Following ingestion, the patient is positioned in front of the image acquisition device, typically an ultra-wide field scanning laser ophthalmoscope (Optos California, Optos PLC, Dunfermline), as this enables fast image acquisition for less cooperative children and visualisation up to 200 degrees of the retinal periphery [5].

**Fig. 2:**
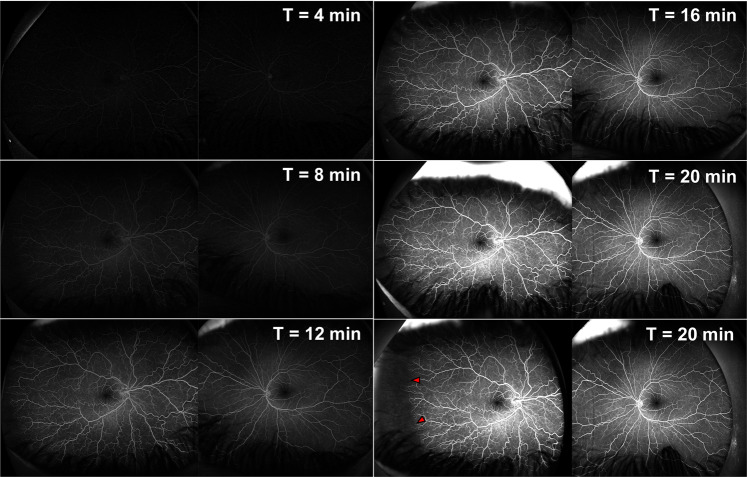
FFA imaging in a patient with abnormal congenital vascular development in the right eye who underwent the oral FFA protocol. Each panel shows the stages of the oral FFA imaging at different time (T) intervals. As can be seen, the choroidal flush and cilioretinal/optic disc vessels show early filling. This is followed by the arterial and capillary phases, as time continues showing the venous phase. In this patient, we can see normal vessel filling and perfusion throughout the FFA imaging in the left eye, in the right eye, marked vascular tortuosity is observed with excessive vessels. In the capillary and venous phases, in the far temporal periphery (Zone 3) there is a large avascular zone (red arrows), with some anastomosis of the retinal vessels.

Our experience is that the time-to-onset using 20% Fluorescein solution typically takes 2–5 min for a choroidal flush to appear shortly followed by retinal circulation. Following this stage, the circulatory filling times are proportionately delayed, but main phases are typically complete within 20 min of consumption for diagnostic purposes (Fig. 2). Adverse effects are rare [2], although mild symptoms may include temporarily stained teeth or a yellow discolouration of skin.

We describe our departments protocol for performing oral FFA in children, a specialised but valuable clinical technique in the evaluation of children with conditions affecting the eye. We hope in future to provide more robust reference timings of major circulatory phases in children undergoing this technique, alongside patient experience to disseminate our findings for other departments interested in adopting this technique.
